# Antitumor Effects and the Compatibility Mechanisms of Herb Pair *Scleromitrion diffusum* (Willd.) R. J. Wang–*Sculellaria barbata* D. Don

**DOI:** 10.3389/fphar.2020.00292

**Published:** 2020-03-20

**Authors:** Li Lu, Sheng Zhan, Xiaohui Liu, Xin Zhao, Xiukun Lin, Huanli Xu

**Affiliations:** ^1^Department of Pharmacology, School of Basic Medical Sciences, Capital Medical University, Beijing, China; ^2^The Second Affiliated Hospital of Southern University of Science and Technology, Shenzhen, China

**Keywords:** *Scleromitrion diffusum* (Willd.) R. J. Wang (HD), *Scutellaria barbata* D. Don (SB), network pharmacology, antitumor, apoptosis

## Abstract

Herb pair *Scleromitrion diffusum* (Willd.) R. J. Wang (HD) and *Scutellaria barbata* D. Don (SB) has been most frequently used for cancer treatment in traditional Chinese medicine. This study aimed to explore the *in vitro* and *in vivo* antitumor effects of HD-SB extract and to elucidate the underlying compatibility mechanisms. HD, SB, and HD-SB extracts were prepared, and the components were detected by ultraperformance liquid chromatography coupled with quadrupole time-of-flight mass spectrometry method. The *in vitro* antitumor effects of various concentrations of these extract were detected on several tumor cell lines using MTS assay. The *in vivo* antitumor effects were evaluated in Panc28 cells–bearing nude mice model. The compatibility mechanisms of herb pair HD-SB were evaluated based on the systems pharmacology strategy and then validated by cellular experiments. HD-SB extract was demonstrated to inhibit the proliferation of the cancer cell lines dose dependently by MTS assay. *In vivo* antitumor effects of HD-SB were much more potent than either of the two single herbs in Panc28 xenograft mice model. A total 29 active ingredients involved in antitumor effects were selected from HD and SB, and the “herb–composition–target–disease” network was constructed. Then, 58 cancer-related targets and 66 KEGG pathways were identified, and PTGS2-, HSP90-, EGFR-, MMP2-, PPARγ-, and GSTP-mediated pathways were predicted to be the antitumor mechanisms of HD-SB. The cellular experiments showed that HD-SB significantly induced cancer cell apoptosis, decreased p-EGFR, HSP90 and bcl-2 expressions, and increased PPARγ, bax, cleaved caspase 3, cleaved PARP, p-AKT, and p-PI3K expressions compared with HD or SB treatment. Our study showed that HD-SB inhibited tumor growth both *in vitro* and *in vivo*, which might be related with apoptosis induction *via* the EGFR/PPARγ/PI3K/AKT pathway.

## Introduction

Despite the wide use of herb formulas in China and other countries, its modernization faced many obstacles due to its multiherb prescription and complexity. Herb pairs, the most basic composition units of multiherb formulas, are a centralized representative of Chinese herbal compatibility. Because of their simplicity and the basic characteristics of complex formulas, herb pairs are thought to play key roles in the investigation of herb compatibility (Wang et al., 2012; Zhou et al., 2019).

In the clinical practices of traditional Chinese medicine (TCM), herb pair *Scleromitrion diffusum* (Willd.) R. J. Wang (also named *Hedyotis diffusa* Willd., HD, Baihuasheshecao in Chinese) and *Scutellaria barbata* D. Don (SB, Banzhilian in Chinese) is frequently used in numerous classic formulas such as *Erbanerbai Tang*, *Zhongliuhefang*, *Fuzhenghuadu Tang*, *Yupengqianbailian Heji*, and so on (Pan et al., 2016; Yang et al., 2018).

HD, belonging to the Rubiaceae family, is a well-known traditional Chinese medicinal herb. It has long been utilized as a critical ingredient of several TCM formulations for clinical cancer treatment. Previous study showed that HD extract could induce apoptosis and inhibit the proliferation by affecting multiple intracellular targets (Lin et al., 2013; Lin et al., 2015; Sun et al., 2016). Lin et al. (2013) showed HD extract inhibits the growth of colorectal cancer *via* inhibiting tumor angiogenesis by Hedgehog signaling pathway. It also induced the cell apoptosis *via* the interleukin 6–inducible STAT3 pathway (Lin et al., 2015). It was also shown that HD inhibited proliferation and induced apoptosis in colorectal cancer cells *via* regulating PI3K/AKT pathway (Li et al., 2018). SB has long been used in TCM to treat bacterial infections, hepatitis, and tumors along with other herbs (Zhang et al., 2017). SB extract was reported to have anticancer activities in many tumor cell lines, including breast cancer, colorectal cancer, and hepatocarcinoma (Tao and Balunas, 2016; Liu et al., 2018; Wang et al., 2019). SB extract treatment significantly suppressed the activation of several colorectal cancer-related pathways, including STAT3, Erk, and p38 signalings in tumor tissues, and altered the expression of multiple critical target genes such as Bcl-2, Bax, cyclin D1, CDK4, and p21 (Lin et al., 2014). It was also shown that SD inhibited colorectal cancer growth *via* suppression of Wnt/β-catenin signaling pathway (Wei et al., 2017). HD-SB combination has long been used as a classic herb pair for cancer treatment, and clinical practices show that HD-SB combination exhibits better therapeutic effects than the two single individuals (Yeh et al., 2014). However, the compatibility mechanisms of paired HD-SB remain unknown. In this study, the *in vitro* and *in vivo* antitumor effects of HD-SB extract were evaluated, and the compatibility mechanisms were investigated.

## Materials and Methods

### Reagents

Human Bel7402, Panc28 cells were obtained from the cell bank of Chinese Academy of Sciences Shanghai (Shanghai, China). Female C57BL/6 mice (6–8 weeks old) were purchased from the Beijing Vital River Laboratory Animal Technology Co. (Beijing, China). Asperulosidic acid methyl ester, kaempferol, rutin, quercetin, apigenin, scutellarin, luteolin, quercetin, and scutebarbatine B were bought from Chroma Biotechnology Co. Ltd. (Chengdu, China). 3-(4,5-Dimethylthiazol-2-yl)-5-(3-carboxymethoxyphenyl)-2-(4-sulfophenyl)- 2H- tetrazolium (MTS) assay kit, RIPA lysis buffer, and Annex V/PI apoptosis kit were purchased from Promega (Madison, WI, USA). Antibodies against prostaglandin G/H synthase 2 (PTGS2), phosphorylated epidermal growth factor receptor (p-EGFR), EGFR, p-AKT, p-PI3K, peroxisome proliferator-activated receptor γ (PPAR**γ)**, glutathione S–transferase P (GSTP), and GAPDH were purchased from Abcam (Cambridge, MA, USA). Antibodies for Bcl-2, bax, cleaved caspase 3, cleaved PARP, heat shock protein 90 (HSP90), and 72-kDa type IV collagenase (MMP2) were purchased from Santa Cruz (Dallas, TX, USA). Dulbecco modified Eagle medium, RPMI1640 medium, and fetal bovine serum were purchased from HyClone (Logan, UT, USA). Mammalian whole-cell protein extraction kit and BCA protein assay kit were bought from Thermo Fisher Scientific (Rockford, IL, USA).

### Preparation of HD-SB

HD and SB herb were purchased from Beijing Tongren Co., Ltd. (batch no. 1711046, 1704050, Beijing, China) and identified by Dr. Rui He (School of Traditional Chinese Medicine, Capital Medical University). Voucher specimens (ID: 2018-EJ024, 2018-EJ025, 2018-EJ026) were deposited in the Department of Materia Medica, School of Chinese Medicine, Capital Medical University, China. The dried HD and SB were washed and pulverized into powder. HD, SB, and HD-SB (1:1 wt/wt) were weighed and extracted with 10× volume 70% ethanol for 2 h using heating reflux at 100°C and repeated for three times. The extract solution was filtered and concentrated using vacuum evaporation, and the residual solution was then freeze dried and stored at 4°C. Because the major components of SB and HD are ﬂavonoids, total contents of ﬂavonoids were used as the quality assessment of the extract using the method in Pharmacopoeia of the People's Republic of China (Chinese Pharmacopoeia Commission, 2015). Total ﬂavonoids in SB were expressed as microgram scutellarin equivalents/100 g. Total ﬂavonoids in HD were expressed as microgram quercetin equivalents/100 g.

### Ultraperformance Liquid Chromatography/MS Analysis

Component analysis of the extracts of HD, SB, and HD-SB was performed on ultraperformance liquid chromatography (UPLC) coupled with quadrupole time-of-flight mass spectrometry (MS) system (Waters Corporation, Milford, MA, USA). HD, SB, and HD-SB extract were weighed accurately and dissolved into 10 mg/mL in methanol for analysis. Asperulosidic acid methyl ester, kaempferol, rutin, quercetin, apigenin, scutellarin, luteolin, quercetin, and scutebarbatine B were used as chemical standard compounds. Standard solutions were prepared by dissolving to 1 mg/mL in methanol. The samples were separated by a Waters ACQUITY UPLC HSS T3 Column (2.1 × 100 mm, 1.8 µm; Waters Corporation). The mobile phase consisted of gradient mixture of acetonitrile (A) and 0.1% formic acid in water (B): 0 to 15 min, 95% to 80% A (vol/vol); 15 to 25 min, 80% to 50% A (v/v); 25 to 30 min, 50% to 20% A (vol/vol). The flow rate was 0.3 mL/min, and the detector scanned from 200 to 400 nm. The full-scan LC-MS data were acquired in both positive and negative ion modes from 30 to 1,300 Da with a 0.4-s scan time. The data were collected, and chromatogram was processed with MassLynx V4.1 software (Waters Corp). Molecular formula speculations of the compounds were determined with Elemental Composition software (Waters Corporation, Milford, MA, USA). Structural identification of the main compounds was determined with Mass Fragment software (Waters Corporation, Milford, MA, USA).

### MTS Assay

The effects of HD, SB, and HD-SB extracts on the growth of different cancer cell lines were detected by MTS assay. Briefly, cells were seeded in 96-well plates and incubated with various concentrations of HD, SB, and HD-SB extracts (0, 12.5, 25, 50, 100, 200 μg/mL). After 48-h incubation, the medium was replaced with new medium containing MTS and incubated for 2 h at 37°C. Then, the Optical Densisty (OD) value was measured at 490 nm using a microplate reader (EXL800; BioTek, Winooski, VT, USA). The IC_50_ value (the half maximal inhibitory concentration) was defined as the concentration of drug inhibiting 50% of cells.

### *In Vivo* Antitumor Evaluation in Tumor-Bearing Nude Mice Model

The animal experiments were approved by the Committee of Animal Experiments and Experimental Animal Welfare of Capital Medical University in Beijing, China (no. AEEI-2019-078), and performed in accordance with the local institutional guidelines and ethics.

Panc28 cells in logarithmic growth phase were harvested and adjusted into the concentration of 1 × 10^7^cells/mL. Female BALB/c nude mice (4–6 weeks, 18–20 g) were inoculated with 0.2 mL of the cell suspension subcutaneously. When the tumor grew up to approximately 100 mm^3^, the mice were randomly divided into the following groups (n = 5): negative control group (0.9% normal saline), 150 mg/kg HD group, 150 mg/kg SB group, 150 mg/kg HD-SB group, or positive control group [50 mg/kg, cyclophosphamide (CTX)], respectively. The drugs were given intragastrically for three times a week. Tumor sizes and body weights were measured every day for 2 weeks. The tumor volumes were calculated according to the following formula: tumor volume = length × width^2^ × 0.5. All mice were sacrificed at the end of the experiment, and tissues were collected for analysis.

The excised tumor tissues were fixed with 4% paraformaldehyde and cut into 4- to 6-mm-thick sections. Then the tissue sections were stained with hematoxylin and eosin. Images were taken with a high-capacity digital slide scanner system (3DHISTECH Ltd., Budapest, Hungary).

### Network Pharmacology Analyses

#### Chemical Ingredients Database Building and Target Prediction

Information on 124 active components related to HD and SB was collected from the Traditional Chinese Medicine Systems Pharmacology Database and Analysis Platform (TCMSP, http://tcmspw.com/tcmsp.php). Considering HD and SB are orally administrated in cancer treatment, the principle for screening potential active ingredients was deﬁned as follows: oral bioavailability (OB) ≥30% and drug-likeness (DL) ≥ 0.18.

Then, ingredients and targets interactions were obtained from TCMSP, and only the proteins that had direct interactions with each chemical in HD and SB were selected as the putative targets.

#### Network Construction and Pathway Analysis

To investigate the action mechanism of active compounds of HD and SB against cancer, the Cytoscape 3.6.1 software package (Boston, MA, USA) was used to construct the “herb–composition–target–disease” network. Two important parameters, degree (the number of edges connected to the node) and betweenness (the number of times a node acts as a bridge along the shortest path between two other nodes), were calculated by Network Analyzer of Cytoscape 3.6.1.

Gene Ontology (GO) enrichment analysis was performed using BiNGO of Cytoscape 3.6.1. The false discovery rate (FDR) was introduced to reveal a multiple-hypothesis testing faulty measure of *p* values, and only GO terms with *p* < 0.01 were chosen. FDR (*p* < 0.05) was employed as an important cutoff in the analysis.

Pathway enrichment analyses using Kyoto Encyclopedia of Genes and Genomes (KEGG, http://www.genome.jp/kegg/) were performed, and significant pathways were evaluated using the Database Visualization and Integrated Discovery system (DAVID, http://david.abcc.ncifcrf.gov/home.jsp, version 6.8) (Yu et al., 2018). The threshold parameters were set at ease = 0.05, count = 5.

### Apoptosis Analysis

Annexin V–fluorescein isothiocyanate (FITC)/propidium iodide (PI) staining kit was used for detecting apoptosis rates induced by HD, SB, and HD-SB according to the manufacturer's instruction. Briefly, after treatment with HD, SB, and HD-SB for 48 h, the cells were collected and washed three times with washing buffer. Then, approximately 1 × 10^5^ cells were resuspended in 500 μL detection buffer. Finally, 5 μL of PI and 5 μL of annexin V-FITC were added and analyzed using a flow cytometry (BD FACSCanto™, San Diego, CA, USA).

### Western Blot

After treatment with HD, SB, and HD-SB for 48 h, cells were collected by centrifugation at 3,000 rpm for 10 min. Whole-cell proteins were extracted using mammalian whole-cell protein extraction kit (Promega, Beijing, China). Supernatant protein concentrations were determined using BCA protein assay. Equal amount of protein was loaded and separated on 10% sodium dodecyl sulfate–polyacrylamide gel electrophoresis before being transferred to polyvinylidene fluoride membranes. The membranes were then incubated with 5% nonfat milk for 2 h. After overnight incubations with primary antibodies at 4°C, the membranes were incubated with goat anti–rabbit or goat anti–mouse alkaline phosphatase–conjugated secondary antibodies (1:3,000; Promega). The membranes were detected by enhanced chemiluminescence Western blot detection regents. The immunoreactive bands were analyzed with Image J 1.43 software (National Institutes of Health, Bethesda, MD, USA).

### Statistical Analysis

All data are presented as the mean ± SD of three independent experiments. Two-tailed Student *t* test or analysis of variance by GraphPad Prism 5.0 (GraphPad Software, San Diego, CA, USA) was used for statistical analysis. Differences were considered statistically significant when *p* < 0.05.

## Results

### Components Analysis for HD-SB

Because the main active components of SB and HD were reported to be ﬂavonoids (Wang et al., 2008; Yang et al., 2018), total contents of ﬂavonoids were determined. The total ﬂavonoids in SB are approximately 1.84 g scutellarin equivalents/100 g and that in HD is approximately 2.01 g quercetin equivalents/100 g. The total ﬂavonoids in HD-SB are approximately 2.53 g scutellarin equivalents/100 g and 1.97 quercetin equivalents/100 g.

**Figure 1:**
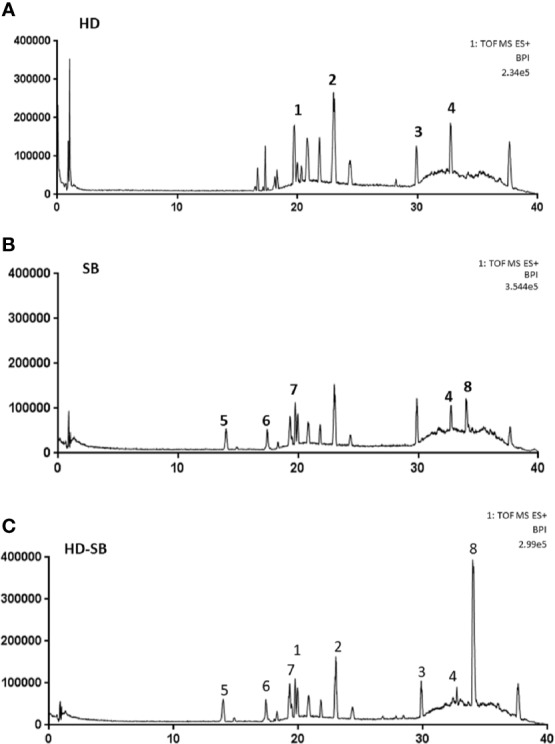
Representative chromatogram by UPLC-MS of *Scleromitrion diffusum* (Willd.) R. J. Wang (HD), *Scutellaria barbata* D. Don (SB), and HD-SB. Results are shown with matching standards of asperulosidic acid methyl ester, kaempferol, rutin, quercetin, apigenin, scutellarin, luteolin, quercetin, and scutebarbatine B. **(A)** HD, **(B)** SB, **(C)** HD-SB.

The main components in HD, SB, and HD-SB were also determined by UPLC-MS. Representative chromatograms by UPLC-MS of SB, HD, and HD-SB are shown in **Figure 1**. Using the standards of main components in SB and HD, we identified that the ingredients in HD were asperulosidic acid methyl ester (retention time, 19.74 min), kaempferol (retention time:24.39 min), rutin (retention time, 29.88), and quercetin (retention time, 32.74 min) (**Figure 1A**), and the main ingredients in SB were apigenin (retention time, 13.98 min), scutellarin (retention time, 17.42 min), luteolin (retention time, 19.31 min), quercetin (retention time, 32.74 min), and scutebarbatine B (retention time, 33.98 min) (**Figure 1B**). All these ingredients can be found in the chromatogram of HD-SB (**Figure 1C**).

### HD-SB Significantly Inhibited Cancer Cell Growth

**Figure 2:**
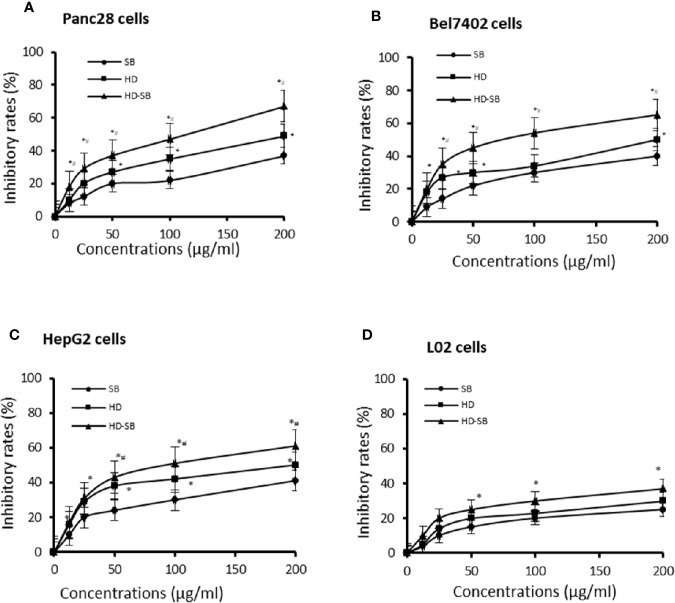
*Scleromitrion diffusum* (Willd.) R. J. Wang (HD), *Scutellaria barbata* D. Don (SB), and HD-SB extract inhibited the cancer cell growth. Panc28 **(A)**, Bel7402 **(B)**, HepG2 **(C)**, and L02 **(D)** cells were treated with different concentrations of HD, SB, and HD-SB for 48 h, and the cell viability was determined by MTS assay. *P < 0.05, compared with the SB group; ^#^*P* < 0.05, compared with the HD group.

The inhibitory effects of SB, HD, and HD-SB on the growth of tumor cell lines (Panc28, Bel7402, and HepG2 cells) and human normal liver cells (L02 cells) were evaluated by MTS assay. As shown in **Figure 2**, SB, HD, and HD-SB inhibited the growth of Panc28, Bel7402, and HepG2 cells dose dependently, and HD-SB showed more obvious inhibitory effects than SB or HD treatment. The IC_50_ of HD-SB on Panc28, Bel7402, and HepG2 cells were 110.50 ± 7.10, 89.48 ± 10.24, and 105.45 ± 9.47 µg/mL, respectively. The IC_50_ of HD on Panc28, Bel7402, and HepG2 cells were 207.14 ± 10.12, 179.35 ± 9.14, and 205.54 ± 9.74 µg/mL, respectively. The IC_50_ of SB on Panc28, Bel7402, and HepG2 cells were all >200 µg/mL. Also, the inhibitory effects of SB, HD, and HD-SB on L02 cells were much weaker than that on cancer cells, with all the IC_50_ > 200 µg/ml.

### HD-SB Significantly Inhibited Cancer Growth *In Vivo*

**Figure 3:**
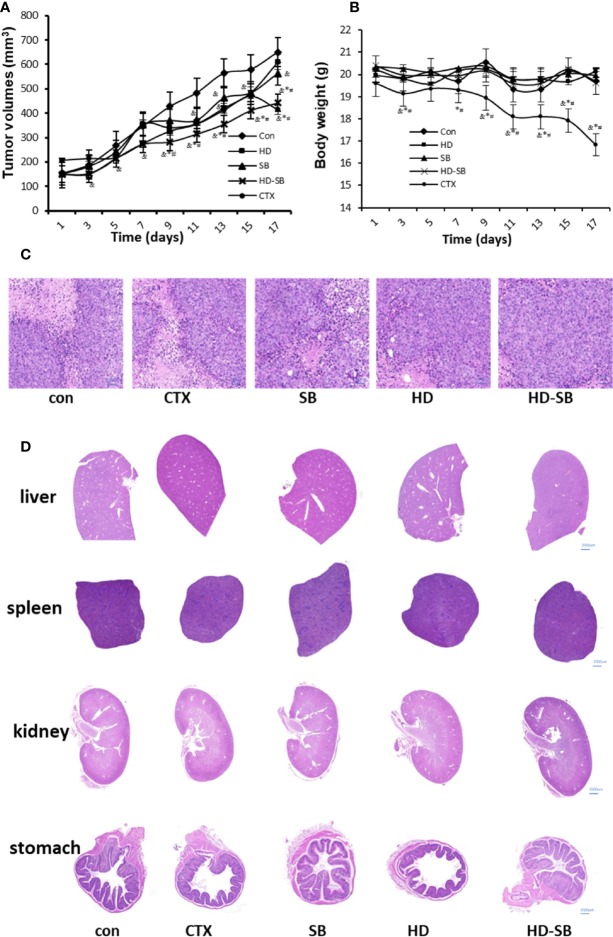
*Scleromitrion diffusum* (Willd.) R. J. Wang (HD)-*Scutellaria barbata* D. Don (SB) extract inhibited tumor growth in the xenograft model. Mice bearing Panc28 cancer cells were treated with HD (150 mg/kg), SB (150 mg/kg), and HD-SB (150 mg/kg). **(A)** Tumor volume measured every 2 days. **(B)** Mouse body weight measured every 2 days. ^&^*P* < 0.05, compared with the control group; *P < 0.05, compared with the SB group; ^#^*P* < 0.05, compared with the HD group. **(C)** Hematoxylin and eosin staining for tumor tissues excised. Scale bar, 200 µm. **(D)** Histological analysis of organs excised from Panc28 tumor-bearing mice, Scale bar, 2,000 µm.

Panc28 cells xenograft tumors in BALB/c nude mice were established and treated with SB, HD, and HD-SB intragastrically. As shown in **Figure 3**, HD-SB treatment displayed more obvious inhibitory effects on tumor growth as compared to HD or SB treatment; the final inhibitory rates of tumor growth were 28.32%, 39.95%, and 52.28%, respectively, in mice treated with SB, HD, or HD-SB. Importantly, the body weight of the mice was less affected in mice treated with SB, HD, or HD-SB, compared with those treated with CTX (**Figure 3B**). Hematoxylin and eosin staining was performed to further evaluate the toxicity of SB, HD, or HD-SB (**Figure 3C**). As shown in **Figure 3D**, no obvious lesions were found in the main organs of mice treated with SB, HD, or HD-SB. These results suggested that HD-SB displayed potent antitumor effect *in vivo* with low toxicity.

### Network Pharmacology Analyses for HD-SB

#### Active *Ingredients* Screening

A total of 131 chemical ingredients of HD and SB were retrieved from TCMSP. The compounds' ADME properties including OB (≥30%) and DL (≥0.18) were used to select active ingredients. As a result, 37 active ingredients were selected. According to the target-disease prediction system in TCMSP, 29 active ingredients were found to have potential anticancer effects among the 37 active ingredients selected. The information of the 29 active components in HD-SB is shown in **Supplementary Table 1**.

#### Target and Function Analysis

To obtain the tumor-related targets of the 29 active components, components-related targets and targets-related disease were further screened using TCMSP. The proteins that had direct interactions with each chemical in HD and SB were selected as the putative targets. After comparison with UniProt Knowledgebase, 54 tumor-related targets were ﬁnally retrieved (**Supplementary Table 2**).

#### Network and Pathway Analysis

**Figure 4:**
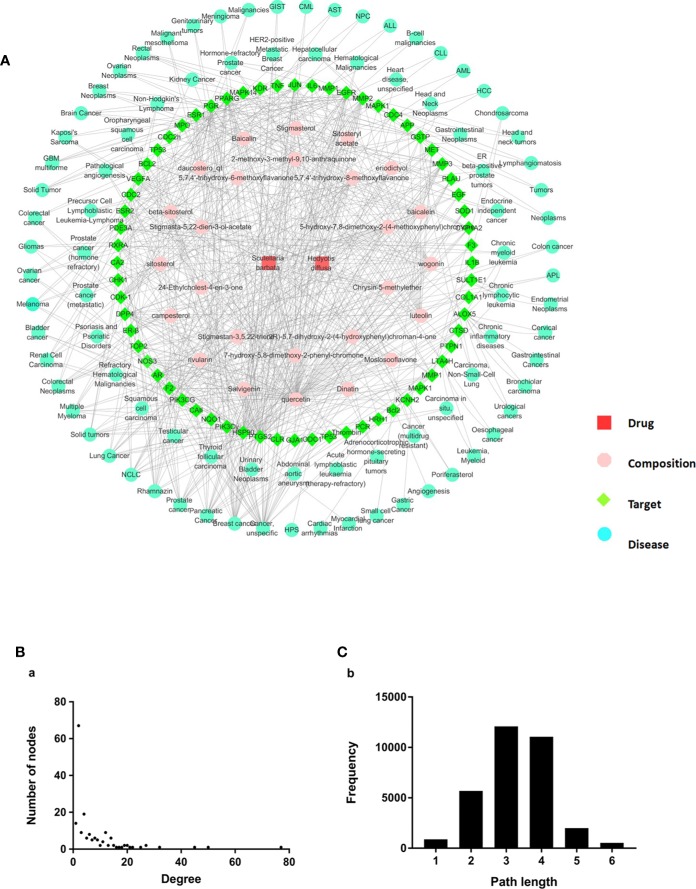
Construction and properties analysis of “herb–composition–target–disease” network of *Scleromitrion diffusum* (Willd.) R. J. Wang (HD) and *Scutellaria barbata* D. Don (SB). **(A)** Herb-composition-target-disease network of HD and SB. **(B)** node degree; **(C)** betweenness distribution (shortest path length distribution).

To investigate the action mechanism of active compounds of HD and SB against cancer, “herb–composition–target–disease” network was constructed using the Cytoscape 3.6.1 software package. As shown in **Figure 4A**, 172 nodes (2 herbs, 29 compounds, 54 targets, and 87 pathways) and 612 edges (relationship between the nodes) were included in the “herb-composition-target-disease.” Detailed information about nodes and parameters of the network are shown in **Supplementary Table 3**. The node degree and betweenness distribution (shortest path length distribution) are shown in **Figures 4B, C**. In the network, nodes and parameters of targets with the degree value ≥10 are shown in **Supplementary Table 4**. And nodes and parameters of diseases with the degree value ≥5 are shown in **Supplementary Table 5**.

The above information suggests that (1) quercetin, luteolin, wogonin, and cesterol (with higher degree) might be the material basis of the antitumor effects of HD-SB; (2) among targets with the degree value ≥10, the top six targets were PTGS2, HSP90, EGFR, MMP2, PPARγ, and GSTP, with the degree values of 50, 45, 27, 25, 22, and 21, respectively, indicating that these targets might be the antitumor targets of HD-SB; (3) among the tumor-related diseases with the degree value ≥5, the top six tumor-related diseases were cancer (unspecific), breast cancer, pancreatic cancer, prostate cancer, non–small cell lung cancer, and solid tumors.

**Figure 5:**
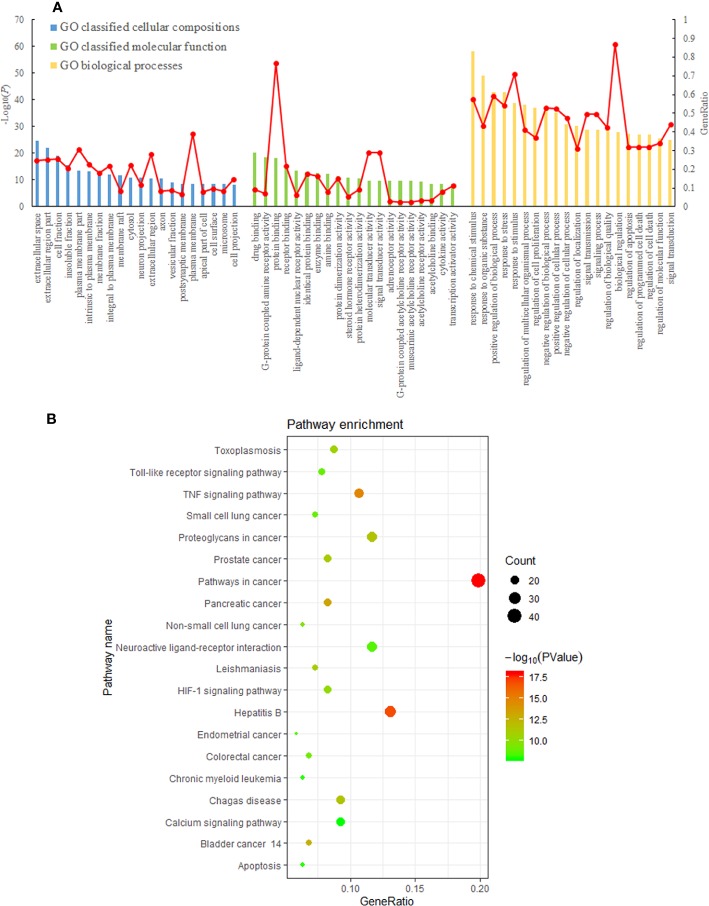
Results of gene ontology (GO) and Kyoto Encyclopedia of Genes and Genomes (KEGG) pathway enrichment analysis. **(A)** Result of GO classified cellular compositions, molecular function, and biological process of *Scleromitrion diffusum* (Willd.) R. J. Wang (HD) and *Scutellaria barbata* D. Don (SB)–related genes. **(B)** KEGG pathway enrichment analysis of *Hedyotis diffusa*–*Sculellaria barbata*–related genes (the first 20 pathways with ease < 0.05, count ≥ 10, and FDR < 0.01).

The GO enrichment analysis included 1,732 GO items, among which 1,450 were related to biological processes, 177 were related to molecular functions, and 105 were related to cell composition (**Figure 5A**). GO classified cellular compositions of HD-SB–related genes, mainly including perikarya, muscle fiber membrane, postsynaptic membrane, mitochondrial outer membrane, vesicles, spindle apparatus, and so on. GO classified molecular function of HD-SB–related genes, mainly including protein kinases binding, protease binding, lipid binding, HSP90 binding, steroid binding, transcriptional factors activation, acetylcholine receptor activity, adrenergic receptor activity, and so on. GO classified biological process of HD-SB–related genes, mainly including cellular process regulation, polymer metabolism regulation, intracellular metabolism regulation, signal transduction, esterase activity regulation, cell proliferation regulation, cell apoptosis regulation, programmed cell death, and so on.

The enrichment analysis of KEGG pathways included 66 KEGG pathways. The top 20 pathways with ease <0.05, count ≥10, FDR <0.01 are shown in **Figure 5B**.

### HD-SB Induced Apoptosis by Targeting EFGR or PPARγ-Mediated Pathways

**Figure 6:**
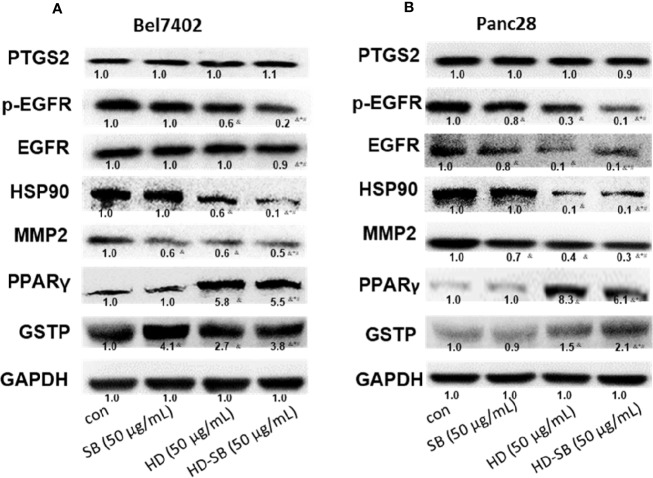
Western blot analysis for the expressions of predicted targets of *Scleromitrion diffusum* (Willd.) R. J. Wang (HD) and *Scutellaria barbata* D. Don (SB) by systems pharmacology. **(A)** Bel7402 cells; **(B)** Panc28 cells.

To validate the obtained results in systems pharmacology analysis, further experiment was performed to delineate the molecular mechanism. The expressions of predicted targets were determined by Western blot analysis. As shown in **Figure 6**, p-EGFR and HSP90 were significantly down-regulated, whereas PPARγ and GSTP were significantly up-regulated after HD-SB treatment, compared with HD or SB treatment.

**Figure 7:**
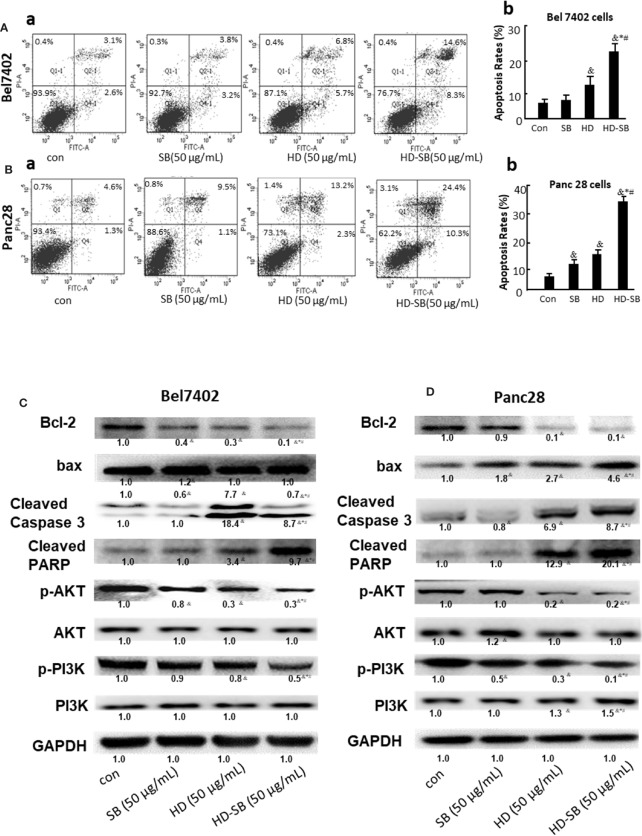
Effects of *Scleromitrion diffusum* (Willd.) R. J. Wang (HD), *Scutellaria barbata* D. Don (SB), and HD-SB extract on apoptosis and expressions of apoptosis-related proteins. Apoptosis was detected by flow cytometry for **(Aa)** Bel7402 cells and **(Ba)** Panc28 cells treated by HD, SB, and HD-SB. Quantification of apoptosis in **(Ab)** Bel7402 cells and **(Bb)** Panc28 cells was shown. ^&^*P* < 0.05, compared with the control group; *P < 0.05, compared with the SB group; ^#^*P* < 0.05, compared with the HD group. Expressions of apoptosis-related proteins in **(C)** Bel7402 cells and **(D)** Panc28 cells treated by HD, SB, and HD-SB were detected by Western blot.

The effects of HD-SB treatment on apoptosis and the proteins involved in apoptosis were also determined. As shown in **Figure 7A**, HD-SB treatment significantly induced apoptosis of both cell lines compared with HD or SB treatment. The apoptosis rates of Bel7402 cells treated with SB, HD, or HD-SB were 7.0%, 12.5%, and 22.9%, respectively, and those of panc28 cells were 10.6%, 15.5%, and 34.7%, respectively. The expressions of apoptosis-related proteins were also determined (**Figure 7B**). The result showed that Bcl-2 expression was obviously decreased, and bax, cleaved caspase 3, and cleaved PARP were obviously increased in the HD-SB treatment group compared with the HD or SB treatment groups. Also, p-AKT and p-PI3K were obviously increased in the HD-SB group compared with HD or SB treatment groups. These results showed that HD-SB inhibited cancer proliferation through induction of apoptosis and suppressed EGFR or PPARγ-related pathways.

## Discussion

Herb pair HD-SB was the core couplet in TCM commonly used for anti-inflammation and anticancer treatments (Pan et al., 2016; Yang et al., 2018). Although HD-SB combination has long been used as a classic herb pair, the bioactive compounds, the potential targets, and compatibility mechanisms of HD-SB remain unknown. In this study, ethanol extract of HD, SB, and equal ratio of HD-SB were prepared. The main components of these extract were determined with the aid of UPLC-MS. The *in vitro* and *in vivo* anti–pancreatic cancer effects of HD-SB extract were evaluated. And the results showed that HD-SB displayed much more potent inhibitory effects on tumor growth compared with HD or SB.

In recent years, network pharmacology analysis was successfully used to predict the underlying function mechanisms of TCM formulas for cancer, depression, and cardiovascular disease treatment (Zhao et al., 2019). Network pharmacology is a systems biology–based methodology for evaluating pharmacokinetics (ADME properties of drugs) and target prediction, as well as investigating the multipharmacological effects of traditional medicines at the molecular level (Di et al., 2018; Gong et al., 2018).

**Figure 8:**
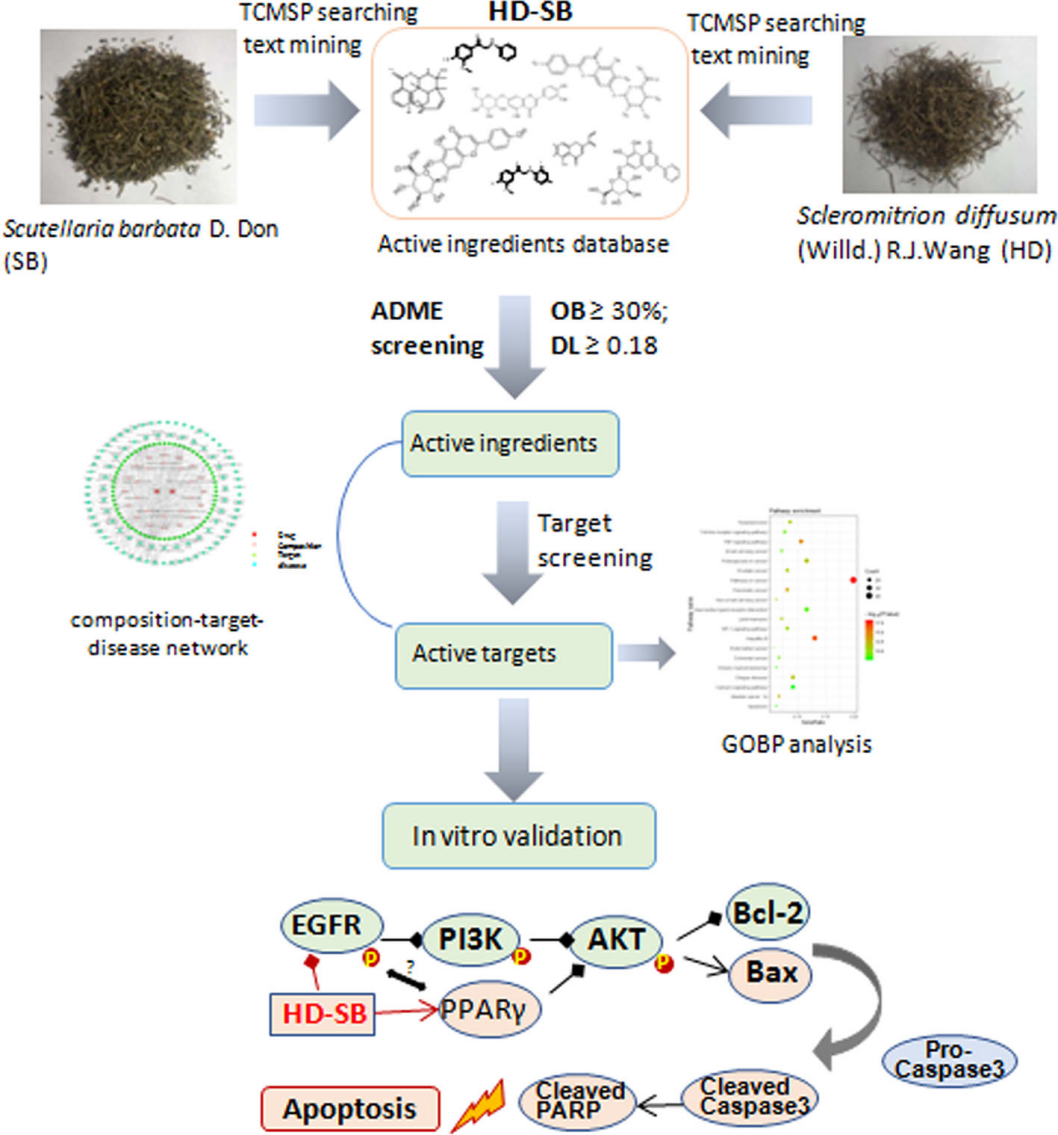
The diagram for studying the compatibility mechanisms of *Scleromitrion diffusum* (Willd.) R. J. Wang (HD), *Scutellaria barbata* D. Don (SB), and (HD-SB).

In the current study, network pharmacology was conducted to predict the potential targets and pathways of HD-SB in cancer treatment. The diagram of this study is shown in **Figure 8**. First, using target-disease prediction system in TCMSP and the screening principle of OB ≥30% and DL ≥0.18, 29 active compounds with potential anticancer effects were obtained. Then “herb–composition–target–disease” network was constructed using Cytoscape 3.6.1 software package, and GO and KEGG pathway enrichment analysis was done. The result showed that (1) quercetin, luteolin, wogonin, and cesterol might be the material basis of the antitumor effects of HD-SB; and (2) PTGS2-, HSP90-, EGFR-, MMP2-, PPARγ-, and GSTP-mediated pathways might be the antitumor mechanism of HD-SB.

To validate the prediction results by network pharmacology analysis, Western blot was conducted to detect the expressions of PTGS2, HSP90, EGFR, MMP2, PPARγ, and GSTP after HD-SB treatment. The result showed that p-EGFR and HSP90 were significantly down-regulated, whereas PPARγ was significantly up-regulated after HD-SB treatment, compared with HD or SB treatment. Because p-EGFR and PPARγ are related with cell apoptosis (Shi et al., 2016; Jackson and Ceresa, 2017), apoptosis rates and apoptosis-related proteins were also determined in cells treated with HD, SB, and HD-SB. It was found that HD-SB treatment significantly decreased bcl-2 expression; increased bax, cleaved caspase 3, cleaved PARP, p-AKT, and p-PI3K; and finally induced cancer cell apoptosis (**Figure 7**).

EGFR is a known oncoprotein, and EGFR ligands activate downstream signaling such as PI3K/AKT, PLCγ/PKC, and STATs signaling cascades, leading to cell proliferation, migration, metastasis, and adhesion (Ni et al., 2017). The PI3K/Akt signaling pathway is a major pathway in regulating cell survival signals. It was reported that PPARγ agonist exhibited antiproliferative activity and induction of apoptosis by inhibiting the PI3K/Akt survival pathway (Hyun et al., 2015). Our present result showed that HD-SB induced apoptosis by activating PPARγ and inhibiting EGFR and PI3K/AKT pathway (**Figure 8**). It was shown that EGFR activation can induce ubiquitination and degradation of PPARγ (Xu et al., 2016). Although several reports show that PPARγ agonists regulate EGFR signaling, almost all of these studies suggest that these effects on EGFR signaling are independent of PPARγ activation (Mansure et al., 2013; Shi et al., 2016). The relationship between EGFR inhibition and PPARγ activation regulated by HD-SB needs further investigation. Also, the mechanisms of EGFR inhibition and PPARγ activation regulated by HD-SB will be further studied.

In summary, our study systematically verified the antitumor effect of HD-SB *in vitro* and *in vivo*. Also, our study showed that HD-SB induced cancer cell apoptosis *via* EGFR/PPARγ/PI3K/AKT pathway and lays a solid foundation for further studies of the herb pair in cancer treatment.

## Data Availability Statement

The raw data supporting the conclusions of this article will be made available by the authors, without undue reservation, to any qualified researcher.

## Ethics Statement

The animal experiments were approved by the Committee of Animal Experiments and Experimental Animal Welfare of Capital Medical University in Beijing, China (No.AEEI-2019-078).

## Author Contributions

LL, XkL, and HX contributed conception and design of the study. LL, SZ, XhL, and XZ performed the study. XZ performed the statistical analysis. HX wrote the first draft of the manuscript. All authors contributed to manuscript revision, read and approved the submitted version.

## Funding

This work was supported by the Natural Science Foundation of China (#81774191), the Beijing Natural Science Foundation (#7172031 and 7172029). This work was also supported by Project of High-level Teachers in Beijing Municipal Universities in the Period of 13th 5-year Plan (#CIT&TCD201804086).

## Conflict of Interest

The authors declare that the research was conducted in the absence of any commercial or financial relationships that could be construed as a potential conflict of interest.
