# Supplementary material for: Antitumor Effects and the Compatibility Mechanisms of Herb Pair Scleromitrion diffusum (Willd.) R. J. Wang–Sculellaria barbata D. Don
Source: Front Pharmacol. 2020 Mar 20;11:292. doi: 10.3389/fphar.2020.00292 (PMC7099881; doi:10.3389/fphar.2020.00292)
Supplement: Supplementary file 1 [file DataSheet_1.docx]

Supplementary Material

Table 1. Information of 29 potential antitumor active components in *Hedyotis diffusa a*nd *Scutellaria barbata*.

| Molecular ID | Compound names | Molecular weight (MW) | Oral bioavailability (OB/%) | drug-likeness (DL) |
| --- | --- | --- | --- | --- |
| MOL005190 | eriodictyol | 288.27 | 71.79 | 0.24 |
| MOL012246 | 5,7,4'-trihydroxy-8-methoxyflavanone | 302.30 | 74.24 | 0.26 |
| MOL012248 | 5-hydroxy-7,8-dimethoxy-2-(4-methoxyphenyl) chromone | 328.34 | 65.82 | 0.33 |
| MOL012251 | Chrysin-5-methylether | 268.28 | 37.27 | 0.20 |
| MOL002714 | baicalein | 270.25 | 33.52 | 0.21 |
| MOL001040 | (2R)-5,7-dihydroxy-2-(4-hydroxyphenyl) chroman-4-one | 272.27 | 42.36 | 0.21 |
| MOL000173 | wogonin | 284.28 | 30.68 | 0.23 |
| MOL000006 | luteolin | 286.25 | 36.16 | 0.25 |
| MOL008206 | Moslosooflavone | 298.31 | 44.09 | 0.25 |
| MOL012250 | 7-hydroxy-5,8-dimethoxy-2-phenyl-chromone | 298.31 | 43.72 | 0.25 |
| MOL012245 | 5,7,4'-trihydroxy-6-methoxyflavanone | 302.30 | 36.63 | 0.27 |
| MOL001735 | Dinatin | 300.28 | 30.97 | 0.27 |
| MOL000098 | quercetin | 302.25 | 46.43 | 0.28 |
| MOL002915 | Salvigenin | 328.34 | 49.07 | 0.33 |
| MOL000351 | Rhamnazin | 330.31 | 47.14 | 0.34 |
| MOL012266 | rivularin | 344.34 | 37.94 | 0.37 |
| MOL000953 | Scutellarin | 462.36 | 37.87 | 0.68 |
| MOL012270 | Stigmastan-3,5,22-triene | 394.75 | 45.03 | 0.71 |
| MOL012254 | campesterol | 400.76 | 37.58 | 0.71 |
| MOL000359 | sitosterol | 414.79 | 36.91 | 0.75 |
| MOL000358 | beta-sitosterol | 414.79 | 36.91 | 0.75 |
| MOL005869 | daucostero_qt | 414.79 | 36.91 | 0.75 |
| MOL002776 | Baicalin | 446.39 | 40.12 | 0.75 |
| MOL000449 | Stigmasterol | 412.77 | 43.83 | 0.76 |
| MOL001755 | 24-Ethylcholest-4-en-3-one | 412.77 | 36.08 | 0.76 |
| MOL001973 | Sitosteryl acetate | 456.83 | 40.39 | 0.85 |
| MOL012269 | Stigmasta-5,22-dien-3-ol-acetate | 454.81 | 46.44 | 0.86 |
| MOL001670 | 2-methoxy-3-methyl-9,10-anthraquinone | 252.28 | 37.83 | 0.21 |
| MOL001659 | Poriferasterol | 412.77 | 43.83 | 0.76 |

Table 2. Targets closely related with tumor and their mapping genes.

| Uniprot | Mapping genes | Tumor related targets |
| --- | --- | --- |
| P06401 | PGR | Progesterone receptor |
| P35354 | PTGS2 | Prostaglandin G/H synthase 2 |
| P08238 | HSP90 | Heat shock protein HSP 90 |
| P48736 | PIK3CG | Phosphatidylinositol-4,5-bisphosphate 3-kinase catalytic subunit, gamma isoform |
| Q12809 | KCNH2 | Potassium voltage-gated channel subfamily H member 2 |
| Q14432 | PDE3A | CGMP-inhibited 3',5'-cyclic phosphodiesterase A |
| P10415 | BCL2 | Apoptosis regulator Bcl-2 |
| P05412 | JUN | Transcription factor AP-1 |
| P03372 | ESR1 | Estrogen receptor |
| P19793 | RXRA | Retinoic acid receptor RXR-alpha |
| P35367 | HRH1 | Histamine H1 receptor |
| P00749 | PLAU | Urokinase-type plasminogen activator |
| P09960 | LTA4H | Leukotriene A-4 hydrolase |
| P10275 | AR | Androgen receptor |
| P37231 | PPARG | Peroxisome proliferator activated receptor gamma |
| P27487 | DPP4 | Dipeptidyl peptidase IV |
| P11388 | TOP2 | DNA topoisomerase II |
| P00734 | F2 | Thrombin |
| P08254 | MMP3 | Matrix metalloproteinase-3 |
| P29474 | NOS3 | Nitric-oxide synthase, endothelial |
| P00533 | EGFR | Epidermal growth factor receptor |
| P15692 | VEGFA | Vascular endothelial growth factor A |
| P08253 | MMP2 | 72 kDa type IV collagenase |
| P01133 | EGF | Pro-epidermal growth factor |
| P01375 | TNF | Tumor necrosis factor |
| P05231 | IL6 | Interleukin-6 |
| P04637 | TP53 | Cellular tumor antigen p53 |
| P11926 | ODC1 | Ornithine decarboxylase |
| P00441 | SOD1 | Superoxide dismutase [Cu-Zn] |
| P03956 | MMP1 | Matrix metalloproteinase-1 |
| P23111 | CDC2 | Cell division control protein 2 homolog |
| P37231 | PPARG | Peroxisome proliferator-activated receptor gamma |
| P05177 | CYP1A2 | Cytochrome P450 1A2 |
| P13726 | F3 | Tissue factor |
| P17302 | GJA1 | Gap junction alpha-1 protein |
| P49888 | SULT1E1 | Estrogen sulfotransferase |
| P02452 | COL1A1 | Collagen alpha-1(I) chain |
| P09917 | ALOX5 | Arachidonate 5-lipoxygenase |
| P05164 | MPO | Myeloperoxidase |
| P09211 | GSTP1 | Glutathione S-transferase P |
| P15559 | NQO1 | NAD(P)H dehydrogenase [quinone] 1 |
| P07339 | CTSD | Cathepsin D |
| P00918 | CA2 | Carbonic anhydrase II |
| P24941 | CDK2 | Cell division protein kinase 2 |
| O14757 | CHEK1 | Serine/threonine-protein kinase Chk1 |
| Q16539 | MAPK14 | Mitogen-activated protein kinase 14 |
| P35968 | KDR | Vascular endothelial growth factor receptor 2 |
| P28482 | MAPK1 | Mitogen-activated protein kinase 1 |
| P11802 | CDK4 | Cell division protein kinase 4 |
| P05067 | APP | Amyloid beta A4 protein |
| P08581 | MET | Hepatocyte growth factor receptor |
| Q92731 | ESR2 | Estrogen receptor beta |
| P01584 | IL1B | Interleukin-1 beta |
| P18031 | PTPN1 | mRNA of Protein-tyrosine phosphatase, non-receptor type 1 |

Table 3. Nodes and parameters of “drug-composition-target- disease” network of Hedyotis diffusa-Sculellaria barbata (Active components).

| Active components | Degree | BC |
| --- | --- | --- |
| quercetin | 77 | 0.4122 |
| luteolin | 20 | 0.0953 |
| wogonin | 19 | 0.0512 |
| beta-sitosterol | 17 | 0.0218 |
| 7-hydroxy-5,8-dimethoxy-2-phenyl-chromone | 14 | 0.0255 |
| 5-hydroxy-7,8-dimethoxy-2-(4-methoxyphenyl) chromone | 12 | 0.0311 |
| baicalein | 12 | 0.0182 |
| Moslosooflavone | 12 | 0.0168 |
| Rhamnazin | 12 | 0.0214 |
| rivularin | 12 | 0.0162 |
| Stigmasterol | 12 | 0.0259 |
| Chrysin-5-methylether | 11 | 0.0132 |
| 5,7,4'-trihydroxy-8-methoxyflavanone | 10 | 0.0059 |
| 2-methoxy-3-methyl-9,10-anthraquinone | 8 | 0.0279 |
| Dinatin | 7 | 0.0047 |
| Salvigenin | 7 | 0.0043 |
| eriodictyol | 5 | 0.0052 |
| (2R)-5,7-dihydroxy-2-(4-hydroxyphenyl) chroman-4-one | 5 | 0.0055 |
| 5,7,4'-trihydroxy-6-methoxyflavanone | 3 | 0.0010 |
| CLR | 2 | 0.0003 |
| Stigmastan-3,5,22-triene | 2 | 0.0003 |
| campesterol | 2 | 0.0003 |
| sitosterol | 2 | 0.0003 |
| daucostero_qt | 2 | 0.0003 |
| Baicalin | 2 | 0.0015 |
| 24-Ethylcholest-4-en-3-one | 2 | 0.0003 |
| Sitosteryl acetate | 2 | 0.0003 |
| Stigmasta-5,22-dien-3-ol-acetate | 2 | 0.0003 |
| Poriferasterol | 2 | 0.0002 |

Table 4. Nodes and parameters of “drug-composition-target- disease” network of Hedyotis diffusa-Sculellaria barbata (Degree of targets≥10).

| Nodes | Names | Degree | BC |
| --- | --- | --- | --- |
| PTGS2 | Prostaglandin G/H synthase 2 | 50 | 0.1551 |
| HSP90 | Heat shock protein HSP 90 | 45 | 0.1326 |
| EGFR | Epidermal growth factor receptor | 27 | 0.0678 |
| MMP2 | 72 kDa type IV collagenase | 25 | 0.0388 |
| PPARG | Peroxisome proliferator-activated receptor gamma | 22 | 0.0554 |
| GSTP | Glutathione S-transferase P | 21 | 0.0785 |
| CDK2 | Cell division protein kinase 2 | 19 | 0.0817 |
| PIK3CG | Phosphatidylinositol-4,5-bisphosphate 3-kinase catalytic subunit, gamma isoform | 18 | 0.0332 |
| DPP4 | Dipeptidyl peptidase IV | 15 | 0.0221 |
| PGR | Progesterone receptor | 15 | 0.0149 |
| ALOX5 | Arachidonate 5-lipoxygenase | 14 | 0.0506 |
| MMP1 | Interstitial collagenase | 14 | 0.0237 |
| JUN | Transcription factor AP-1 | 14 | 0.0199 |
| CDC2 | Cell division control protein kinase 2 | 12 | 0.0197 |
| MMP3 | Stromelysin-1 | 12 | 0.0104 |
| RXRA | Retinoic acid receptor RXR-alpha | 13 | 0.0120 |
| AR | Androgen receptor | 13 | 0.0100 |
| TP53 | Cellular tumor antigen p53 | 12 | 0.0194 |
| ESR1 | Estrogen receptor | 11 | 0.0185 |
| NOS3 | Nitric-oxide synthase, endothelial | 10 | 0.0138 |

Table 5. Nodes and parameters of “drug-composition-target- disease” network of Hedyotis diffusa-Sculellaria barbata (Degree of diseases ≥5).

| Nodes | Names | BC |
| --- | --- | --- |
| Cancer, unspecific | 32 | 0.0657 |
| Breast cancer | 20 | 0.0338 |
| Pancreatic Cancer | 16 | 0.0239 |
| Prostate cancer | 14 | 0.0046 |
| Non-small Cell Lung Cancer | 12 | 0.0112 |
| Solid tumors | 11 | 0.0315 |
| Lung Cancer | 11 | 0.0071 |
| Multiple Myeloma | 8 | 0.0127 |
| Colorectal Neoplasms | 8 | 0.0009 |
| Renal Cell Carcinoma | 7 | 0.0050 |
| Bladder cancer | 6 | 0.0024 |
| Ovarian cancer | 6 | 0.0017 |
| Melanoma | 6 | 0.0008 |
| Hepatocellular carcinoma | 5 | 0.0013 |
